# Assessment of Eccentric Exercise-Induced Oxidative Stress Using Oxidation-Reduction Potential Markers

**DOI:** 10.1155/2015/204615

**Published:** 2015-03-22

**Authors:** Dimitrios Stagos, Nikolaos Goutzourelas, Amalia-Maria Ntontou, Ioannis Kafantaris, Chariklia K. Deli, Athanasios Poulios, Athanasios Z. Jamurtas, David Bar-Or, Dimitrios Kouretas

**Affiliations:** ^1^Department of Biochemistry and Biotechnology, University of Thessaly, Ploutonos 26 & Aiolou, 41221 Larissa, Greece; ^2^Department of Exercise and Sport Sciences, University of Thessaly, 42100 Trikala, Greece; ^3^Trauma Research Department, St. Anthony Hospital, Lakewood, CO 80228, USA; ^4^Trauma Research Department, Swedish Medical Center, Englewood, CO 80113, USA; ^5^Trauma Research Department, Medical Center of Plano, Plano, TX 75075, USA

## Abstract

The aim of the present study was to investigate the use of static (sORP) and capacity ORP (cORP) oxidation-reduction potential markers as measured by the RedoxSYS Diagnostic System in plasma, for assessing eccentric exercise-induced oxidative stress. Nineteen volunteers performed eccentric exercise with the knee extensors. Blood was collected before, immediately after exercise, and 24, 48, and 72 h after exercise. Moreover, common redox biomarkers were measured, which were protein carbonyls, thiobarbituric acid-reactive substances, total antioxidant capacity in plasma, and catalase activity and glutathione levels in erythrocytes. When the participants were examined as one group, there were not significant differences in any marker after exercise. However, in 11 participants there was a high increase in cORP after exercise, while in 8 participants there was a high decrease. Thus, the participants were divided in low cORP group exhibiting significant decrease in cORP after exercise and in high cORP group exhibiting significant increase. Moreover, only in the low cORP group there was a significant increase in lipid peroxidation after exercise suggesting induction of oxidative stress. The results suggested that high decreases in cORP values after exercise may indicate induction of oxidative stress by eccentric exercise, while high increases in cORP values after exercise may indicate no existence of oxidative stress.

## 1. Introduction

Eccentric exercise is an active contraction of a muscle occurring simultaneously with lengthening of the muscle and induces severe muscle damage characterised by decreased muscle force production [1, 2], increased serum creatine kinase (CK) activity [1, 2], and inflammation response [3–5]. Specifically, eccentric exercise induces damage to skeletal muscle in a fiber specific manner [6]. Reactive species (RS) have been shown to play an important role in both the initiation and the progression of muscle fiber injury after eccentric exercise [7, 8]. The generation of RS during an extended bout of eccentric exercise has been attributed to different mechanisms such as xanthine and NADPH oxidase production, ischemia reperfusion, prostanoid and catecholamine metabolism, disruption of iron-containing proteins, and excessive calcium accumulation [9]. Moreover, the infiltration of neutrophils and macrophages to the site of injury [10] after eccentric exercise generate RS [11], which damage the muscle tissue [8].

However, although it is taken as granted that eccentric exercise induces oxidative stress, great differences have been shown in the extent of oxidative stress between different individuals after eccentric exercise [12]. Moreover, Margaritelis et al. [12] have reported that eccentric exercise can induce reductive stress or negligible stress in a considerable number of people. These differences impose the need for finding markers that could predict the severity of oxidative stress induced by eccentric exercise. In our previous studies [13, 14] as well as in other studies, increases in oxidative stress markers have been reported in blood after eccentric exercise [15–17]. For example, eccentric exercise has been shown to increase malondialdehyde (MDA), protein carbonyl (CARB), and F2-isoprostane levels [13, 15, 17] and decrease glutathione levels (GSH) [12, 13]. However, most of these markers assess a specific damage induced by RS, and so there is also the need for markers that could measure the total redox status of an individual after eccentric exercise.

Thus, the aim of the present study was to investigate if markers based on the measurement of oxidation-reduction potential (ORP) can be used for predicting the total oxidative stress induced by eccentric exercise. ORP assessment was made by a new methodology used by the Luoxis' proprietary RedoxSYS Diagnostic System. RedoxSYS Diagnostic System measures ORP which is an integrated measure of the balance between total oxidants (e.g., oxidized thiols, superoxide radical, hydroxyl radical, hydrogen peroxide, nitric oxide, peroxynitrite, and transition metal ions) and total reductants (e.g., free thiols, ascorbate, a-tocopherol, b-carotene, and uric acid). Thus, ORP is an overall measure of the oxidative stress to which a biological system is subjected [18]. The RedoxSYS Diagnostic System enables robust and rapid assessment of oxidative stress in a single drop of plasma via measurement within four minutes of two distinct elements to determine ORP, the static ORP (sORP), and the capacity ORP (cORP). sORP is the standard potential between a working electrode and a reference electrode with no driving current (or extremely small current) which is proportional to the balance of reductants and oxidants and is what is classically termed ORP (i.e., a homeostatic parameter capturing the current balance of oxidants and reductants in a biological specimen). Low sORP values mean that the biological sample is in the normal range of oxidative stress, while higher than normal sORP values mean that the biological sample is in a higher state of oxidative stress. cORP is the measure of antioxidant reserve available in the body's system; high capacity values mean that the biological sample has antioxidant reserves in the normal range; lower than normal cORP values mean that the biological sample has below normal antioxidant reserves. Specifically, the RedoxSYS Diagnostic System measures the ORP with a three-electrode system, a working electrode, a counter electrode, and a reference electrode. First, a negligible amount of current is applied between the working and counter electrodes, and the ORP is measured between the working and reference electrodes. Once the ORP reading reaches equilibrium, the sORP is established and measured in millivolts (mV). Then, a linearly increasing current is applied to the sample, between the counter and working electrodes. The time from the beginning of the current sweep to the maximum rate of change in ORP is referred to as transition time and the integrated current to this time is the cORP, measured in microcoulombs (*μ*C). In one of our previous studies, the RedoxSYS Diagnostic System has been shown to be effective for assessing oxidative stress induced after a marathon race [19].

The redox status of the participants in the present study was also assessed by “conventional” oxidative stress markers such as GSH, catalase activity, thiobarbituric acid-reactive substances (TBARS), CARB, and total antioxidant capacity (TAC). Thus, ORP values were compared with those of “conventional” oxidative stress markers to examine if the former could predict eccentric exercise-induced oxidative stress.

Moreover, two different methodologies were used for examining if ORP markers could be used for the monitoring eccentric exercise-induced oxidative stress. In the first methodology, all the participants were examined as one group before and after exercise. However, there is recently growing evidence that there is a marked heterogeneity in responses to eccentric exercise-induced oxidative stress between different individuals [12]. Similarly, in the present study, it was observed that the participants could be divided into two groups, those with high increase and those with large decrease in cORP after exercise compared to before exercise. Thus, for the analysis of the results a second methodology was followed in which pre- and postexercise comparison for all tested oxidative stress markers was made separately for each of these groups.

## 2. Materials and Methods

### 2.1. Participants

Nineteen young volunteers (gender: 10 men and 9 female; age: 24.4 ± 4.0 years; height: 168.6 ± 7.5 cm; weight: 69.4 ± 4.0 kg) participated in the present investigation. Subjects were excluded from the study, if they had any history of musculoskeletal injury to the lower limbs that would limit the ability to perform the exercise session. Smoking and consumption of nutritional supplementation the last three months before the study initiation were also exclusion criteria. During their first visit, body mass was measured to the nearest 0.5 kg (Beam Balance 710, Seca, United Kingdom) while the subjects were lightly dressed and barefoot. Standing height was measured to the nearest 0.5 cm (Stadiometer 208, Seca). Volunteers were instructed to abstain from any strenuous exercise during their participation in the study as well as for five days prior and 3 days following the exercise session. Subjects were also advised to refrain from taking anti-inflammatory or analgesic medications for the duration of the study. A written consent was obtained from all participants, after they were informed for the risks, discomforts, and benefits involved in the study. The procedures were in accordance with the Helsinki Declaration of 1975, as revised in 2000.

### 2.2. Eccentric Exercise Protocol

The eccentric exercise session was performed on an isokinetic dynamometer (Cybex Norm, Ronkonkoma, NY). The exercise protocols were undertaken from the seated position (120° hip angle) with the lateral femoral condyle aligned with the axis of rotation of the dynamometer. Participants were coupled to the dynamometer by an ankle cuff attached proximal to the lateral malleolus, after they were stabilized according to the manufacturer's instructions. Participants completed 5 sets of 15 eccentric maximal voluntary contractions (knee range, 0° full extension to 90° flexion) at an angular velocity of 60°/s. A 2 min rest interval was used between sets and the total workout time was 15 min. Feedback of the intensity and duration of eccentric exercise was provided automatically by the dynamometer. During the exercise session, the subjects were verbally encouraged to maximally activate their knee extensors, even though, because of fatigue, the performance was declined as the exercise progressed. Before the exercise session, subjects performed an 8 min warm-up consisting of cycling on a Monark cycle ergometer (Vansbro, Sweden) at 70 rpm and 50 W.

### 2.3. Assessment of Muscle Pain

To confirm the presence of delayed onset muscle soreness (DOMS) immediately after exercise and 24, 48, and 72 h after exercise, participants verbally rated on a scale from 0 (“no soreness”) to 10 (“worst soreness imaginable”) their perceived pain during walking (DOMSw) and making the squat movement (DOMSsq).

### 2.4. Muscle Damage

The isokinetic dynamometer was used for the measurement of isometric knee extensor peak torque at 90° knee flexion. The average of the 3 maximal voluntary contractions with the preferred leg was recorded. To ensure that the subjects provided their maximal effort, the measurements were repeated if the difference between the lower and the higher torque values exceeded 10%. There was a 2 min rest between isometric efforts.

### 2.5. Blood Collection and Handling

All participants performed an acute isokinetic eccentric exercise bout with the knee extensors of their preferred leg. Blood samples (10 mL) were drawn from a forearm vein with subjects in a seated position before, immediately after exercise, and 24 h, 48 h, and 72 h after exercise. Blood was collected in ethylenediamine tetraacetic acid (EDTA) tubes for measuring TAC, TBARS, CARB, and GSH levels and catalase activity. Blood was also collected in heparin tubes for measuring ORP. Blood samples were centrifuged immediately at 1370 g for 10 min at 4°C and the plasma was collected and used for the above measurements. The packed erythrocytes were lysed with distilled water (1 : 1 v/v), inverted vigorously, and centrifuged at 4020 g for 15 min at 4°C and the erythrocyte lysate was collected for measurement of catalase activity. A portion of erythrocyte lysate (500 *μ*L) was treated with 5% trichloroacetic acid (TCA) (1 : 1 v/v), vortexed vigorously, and centrifuged at 28,000 g for 5 min at 4°C. The supernatants were removed, treated again with 5% TCA (1.3 : 1 v/v), and centrifuged again at 28,000 g for 5 min at 4°C. The clear supernatants were transferred to eppendorf tubes and were used for the determination of GSH. Plasma and erythrocyte lysate were stored at 80°C prior to biochemical analyses.

### 2.6. Assessment of sORP and cORP Using the RedoxSYS Diagnostic System

sORP and cORP values were determined using the RedoxSYS Diagnostic System (Luoxis Diagnostics, Inc., Englewood, CO, USA). In particular, 20 *μ*L of plasma was applied to disposable sensors, which were inserted into the RedoxSYS Diagnostic System that measured and reported within four minutes the sORP and cORP values. sORP captures the integrated balance of oxidants and reductants in a specimen and is reported in millivolts (mV). cORP is the amount of antioxidant reserves and is expressed in microcoulombs (*μ*C).

### 2.7. Assessment of TAC, TBARS, GSH, Catalase Activity, and CARB

For TBARS determination, a slightly modified assay of Keles et al. [20] was used. According to this method, 100 *μ*L of plasma was mixed with 500 *μ*L of 35% TCA and 500 *μ*L of tris(hydroxymethyl)aminomethane hydrochloride (Tris–HCl) (200 mM, pH 7.4) and incubated for 10 min at room temperature. One milliliter of 2 M Na_2_SO_4_ and 55 mM thiobarbituric acid solution was added and the samples were incubated at 95°C for 45 min. The samples were cooled on ice for 5 min and were vortexed after adding 1 mL of 70% TCA. The samples were centrifuged at 15,000 g for 3 min and the absorbance of the supernatant was read at 530 nm. A baseline absorbance was taken into account by running a blank along with all samples during the measurement. Calculation of TBARS concentration was based on the molar extinction coefficient of MDA. The intra- and interassay coefficients of variation (CV) for TBARS were 3.9% and 5.9%, respectively.

Protein carbonyls were determined based on the method of Patsoukis et al. [21]. In this assay, 50 *μ*L of 20% TCA was added to 50 *μ*L of plasma and this mixture was incubated in an ice bath for 15 min and centrifuged at 15,000 g for 5 min at 4°C. The supernatant was discarded and 500 *μ*L of 10 mM 2,4-dinitrophenylhydrazine (DNPH) (in 2.5 N hydrochloride (HCl)) for the sample, or 500 *μ*L of 2.5 N HCl for the blank, was added to the pellet. The samples were incubated in the dark at room temperature for 1 h, with intermittent vortexing every 15 min and were centrifuged at 15,000 g for 5 min at 4°C. The supernatant was discarded and 1 mL of 10% TCA was added, vortexed, and centrifuged at 15,000 g for 5 min at 4°C. The supernatant was discarded and 1 mL of ethanol-ethyl acetate (1 : 1 v/v) was added, vortexed, and centrifuged at 15,000 g for 5 min at 4°C. This washing step was repeated twice. The supernatant was discarded and 1 mL of 5 M urea (pH 2.3) was added, vortexed, and incubated at 37°C for 15 min. The samples were centrifuged at 15,000 g for 3 min at 4°C and the absorbance was read at 375 nm. Calculation of protein carbonyl concentration was based on the molar extinction coefficient of DNPH. The intra- and interassay CV for protein carbonyls were 4.3% and 7.0%, respectively. Total plasma protein was assayed using a Bradford reagent from Sigma-Aldrich.

GSH was measured according to Reddy et al. [22]. Twenty microliters of erythrocyte lysate treated with 5% TCA was mixed with 660 *μ*L of 67 mM sodium potassium phosphate (pH 8) and 330 *μ*L of 1 mM 5,5′-dithiobis-2 nitrobenzoate (DTNB). The samples were incubated in the dark at room temperature for 45 min and the absorbance was read at 412 nm. GSH concentration was calculated relative to a calibration curve made using commercial standards. The intra- and interassay CV for GSH were 3.1% and 4.5%, respectively.

Catalase activity was determined using the method of Aebi [23]. Briefly, 4 *μ*L of erythrocyte lysate (diluted 1 : 10) was added to 2991 *μ*L of 67 mM sodium potassium phosphate (pH 7.4) and the samples were incubated at 37°C for 10 min. Five microliters of 30% hydrogen peroxide (H_2_O_2_) was added to the samples and the change in absorbance was immediately read at 240 nm for 130 s. Calculation of catalase activity was based on the molar extinction coefficient of H_2_O_2_. The intra- and interassay CV for catalase were 6.2% and 10.0%, respectively.

The determination of TAC was based on the method of Janaszewska and Bartosz [24]. In particular, 20 *μ*L of plasma was added to 480 *μ*L of 10 mM sodium potassium phosphate (pH 7.4) and 500 *μ*L of 0.1 mM 2,2-diphenyl-1-picrylhydrazyl (DPPH) free radical and the samples were incubated in the dark for 30 min at room temperature. The samples were centrifuged for 3 min at 20,000 g and the absorbance was read at 520 nm. The intra- and interassay CV for TAC were 2.9% and 5.4%, respectively. TAC is presented as mmol of DPPH reduced to 2,2-diphenyl-1-picrylhydrazine (DPPH:H) by the antioxidants of plasma.

### 2.8. Statistical Analysis

For statistical analysis, data were analyzed by one-way ANOVA followed by Dunnett's test for multiple pairwise comparisons. Moreover, two different statistical comparisons were used for the analysis of the results from the oxidative stress markers before and after eccentric exercise. In the first statistical comparison, all the participants were examined as one group before and after exercise. In the second statistical comparison, the participants were divided into two groups, those with high increase and those with large decrease in cORP after exercise compared to before exercise. Then, pre- and postexercise comparison for all tested markers was made separately for each of these groups. Correlation between DOMS and oxidative stress markers was examined by Spearman's correlation analysis. The level of statistical significance was set at *P* < 0.05. For all statistical analyses SPSS, version 13.0 (SPSS Inc., Chicago, Ill) was used. Data are presented as mean ± SEM.

## 3. Results and Discussion

### 3.1. Assessment of Muscle Pain and Muscle Damage

Eccentric exercise increased DOMS from 2.6- to 4.6-fold during walking and from 3.2- to 5-fold when making squat movement (Table 1). The increase in DOMS after eccentric exercise is not completely understood but has been suggested to be caused by various biochemical changes after muscle damage rather than a single event of damage [25]. Thus, the main cause of DOMS is structural muscle damages, fundamentally ruptures within the muscle [25]. This muscle damage induces inflammatory response (e.g., chemokine release, activation of inflammatory cells, increase in prostaglandins, and production of arachidonic acid). These inflammatory compounds interact directly with afferent nerves through pain receptors. When the stimuli from the afferent nerves reach medulla and cerebral cortex, muscle soreness is perceived [25]. Other factors suggested to be involved in the physiological mechanism-induced DOMS are lactic acid and nitric oxides [25]. Moreover, although it is clear that RS are produced after eccentric exercise, it is not clear if there is a direct relationship between them and DOMS [26]. It has been proposed that eccentric exercise-induced inflammation may be the main cause of RS production. Specifically, inflammation causes phagocytic cells to migrate to the damaged tissue and exhibit a respiratory burst resulting in the production of RS such as superoxide and hydrogen peroxide [26]. These RS may cause further damage by killing muscle cells [26]. In the present study, the correlation analysis showed that there was a moderate significant correlation (correlation coefficient *r* = 0.648; *P* < 0.01) between DOMSw and sORP at 48 h after exercise and an inverse correlation between DOMSw and cORP at 48 h (*r* = −0.640; *P* < 0.001) and 72 h (*r* = −0.601; *P* < 0.001) after exercise. Moreover, there was a significant moderate inverse correlation (*r* = −0.588; *P* < 0.001) between DOMSw and catalase activity at 72 h after exercise. The positive correlation between DOMSw and sORP and the inverse correlation between DOMSw and cORP or catalase activity suggest that there was an association of DOMS with oxidative stress. However, this association does not mean necessarily that oxidative stress was an etiologic factor of DOMS. To find out the possible causal relationship between DOMS and RS more experiments are needed in which RS would be inhibited by antioxidant supplementation [26].

Moreover, muscle damage induced by eccentric exercise results in an immediate and prolonged reduction in muscle function, most notably a reduction in force-generating capacity [27]. Thus, isometric torque declined significantly (*P* < 0.05) by 15.8% at the end of the exercise session (before: 243.9 ± 55.4 Nm; after: 205.20 ± 57.54 Nm).

### 3.2. Oxidative Stress Markers

**Figure 1:**
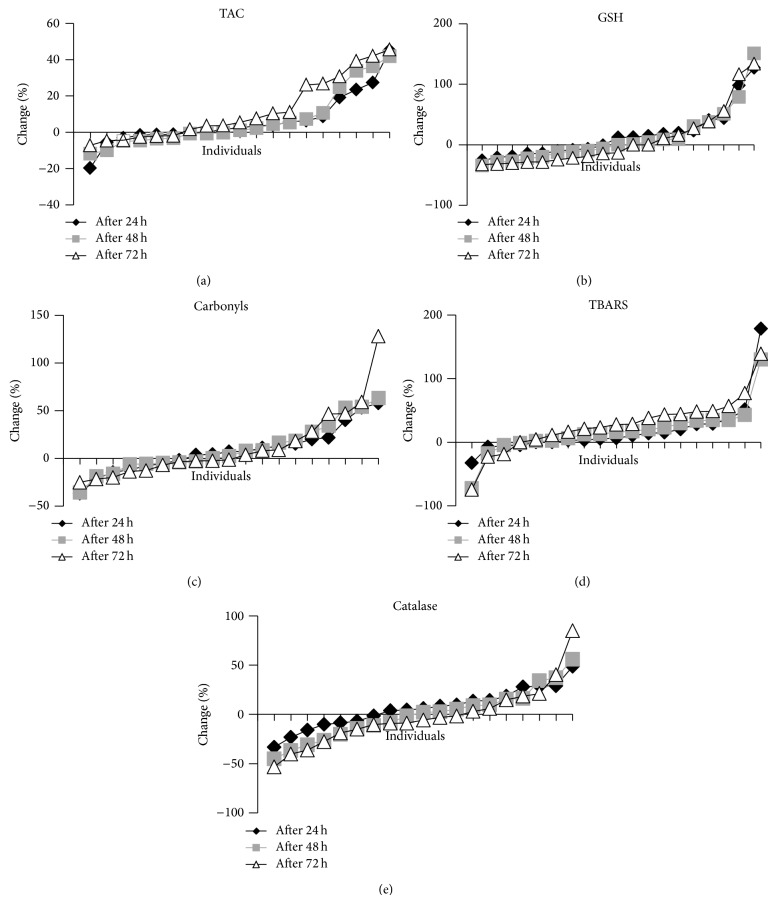
Percent change in redox biomarker levels of each individual at 24 h, 48 h, and 72 h after eccentric exercise. (a) TAC: total antioxidant capacity (in plasma); (b) GSH: reduced glutathione (in erythrocytes); (c) CARB: protein carbonyl levels (in plasma); (d) TBARS: thiobarbituric acid-reactive substances (in plasma); (e) CAT: catalase activity (in erythrocytes).

**Figure 2:**
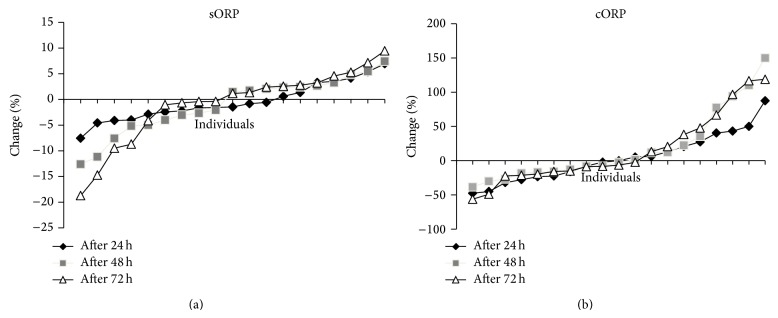
Percent change in ORP marker levels in plasma blood of each individual at 24 h, 48 h, and 72 h after eccentric exercise. (a) sORP: static oxidation-reduction potential; (b) cORP: capacity oxidation-reduction potential.

None of the tested redox markers changed statistically significant postexercise compared to preexercise when all the participants were examined as one group (Table 2). This lack of significance regarding the changes of the oxidative stress markers after exercise can be explained by the great variation that each of them presents between different individuals (Table 2). Moreover, the redox markers in many of the participants in the study changed unexpectedly. For example, in several individuals, TBARS and CARB levels were decreased, while GSH levels were increased after exercise (Figure 1), while according to previous studies the opposite effect was expected [12, 13, 15, 17]. Thus, in all redox markers the participants could be divided into two groups, those with high increase and those with large decrease in the values of the markers after exercise compared to before exercise (Figures 1 and 2). Similar great variation and unexpected findings have also been exhibited in other studies on eccentric exercise [12]. Specifically, Margaritelis et al. [12] have reported that eccentric exercise can induce even reductive stress or negligible stress in a considerable number of people. This great variation in the response of different individuals to eccentric exercise-induced oxidative stress may be attributed to the high complexity of the regulation of redox homeostasis in humans. That is, many different factors such as genetic, physiological, biochemical, and dietary factors can affect the final outcome of oxidant stimuli [28–31].

**Figure 3:**
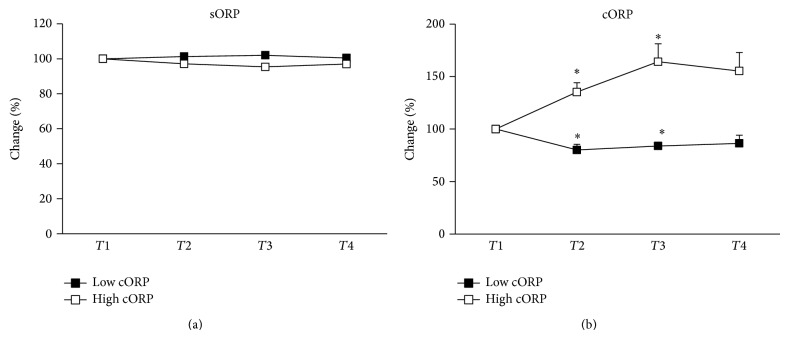
Percentage changes of ORP values of individuals of low cORP and high cORP groups at before exercise (*T*1) and 24 h (*T*2), 48 h (*T*3), and 72 h (*T*4) after eccentric exercise. (a) sORP: static oxidation-reduction potential; (b) cORP: capacity oxidation-reduction potential. ^*^Significantly different compared to preeccentric exercise (*P* < 0.05).

Like all markers, cORP responded differently to eccentric exercise between different individuals. Namely, 42.2% of the participants exhibited high increase in cORP, while the rest 57.8% of the individuals had large decrease in cORP after exercise (Figure 2). As mentioned cORP is an integrated measure of the antioxidant reserve available in the body's system. Thus, the previous observation about the differential response of cORP to eccentric exercise led us to the hypothesis that some of the participants could confront the eccentric exercise-induced oxidative stress by increasing their antioxidant reserves (i.e., increase in cORP). On the other hand, other participants were not able to cope with oxidative stress or to replace their antioxidant reserves after exercise (i.e., decrease in cORP). For testing this hypothesis, the participants were divided in two groups: (i) the first group had high increase in cORP (high cORP group; *n* = 11); (ii) the second group had high decrease in cORP (low cORP group; *n* = 8). In the high cORP group there was statistical significant increase in cORP values at 24 h and 48 h after exercise, while in the low cORP group there was statistical significant decrease in cORP values at 24 h and 48 h after exercise (Figure 3). Afterwards, in these two groups, the change of the other redox markers was examined separately. Although in all markers the participants could be divided in two groups, cORP was selected for this analysis because it is an integrated measure of the total antioxidant capacity of the organism, while most of the other tested markers assess either a specific antioxidant mechanism (e.g., GSH, catalase) or a specific oxidative damage (e.g., TBARS, CARB). TAC also indicates the total antioxidant capacity, but it is based on the reduction of a free radical (i.e., DPPH) by the antioxidant molecules in plasma. Thus, this method is imperfect, since it evaluates only the reductants found in plasma. However, cORP is based on the amount not only of the reductants but also of the oxidants in the plasma. For this reason, cORP measurement may be a more accurate method than TAC as a holistic approach for assessing* in vivo* oxidative stress. Finally, like cORP, sORP measures the overall current redox balance, but this marker in most individuals did not change significantly after exercise and exhibited much less variation than cORP (Figure 2; Table 2).

**Figure 4:**
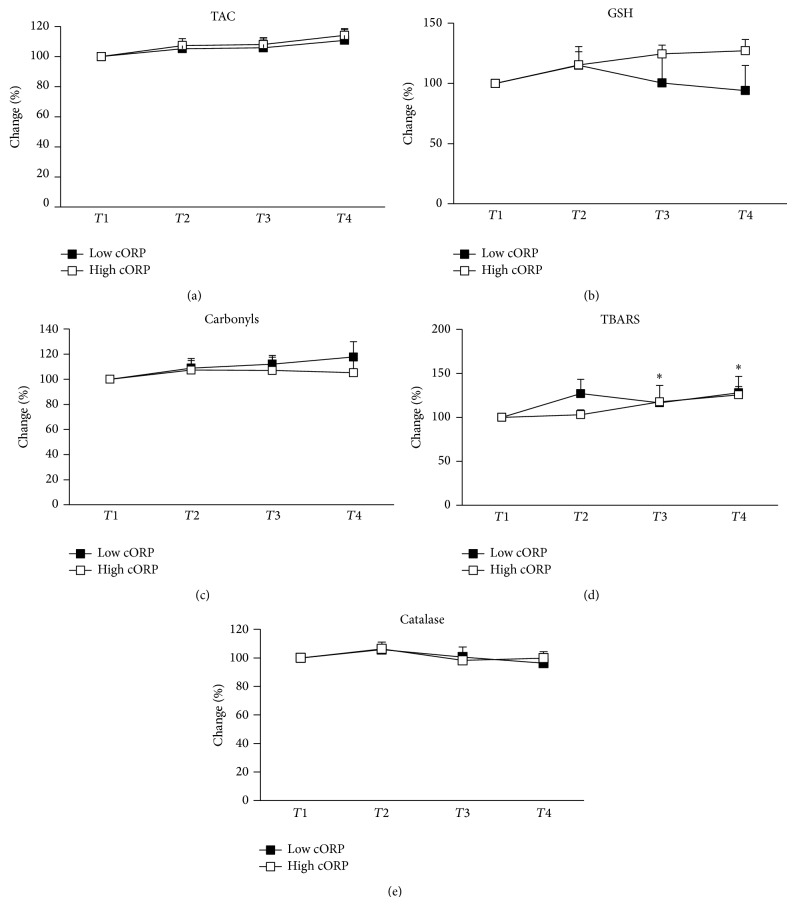
Percentage changes of the redox biomarkers in plasma and erythrocytes of individuals of low cORP and high cORP groups at preexercise (*T*1) and 24 h (*T*2), 48 h (*T*3), and 72 h (*T*4) posteccentric exercise: (a) TAC: total antioxidant capacity; (b) GSH: reduced glutathione; (c) CARB: protein carbonyl levels; (d) TBARS: thiobarbituric acid-reactive substances; (e) CAT: catalase activity. ^*^Significantly different compared to preeccentric exercise in low cORP group (*P* < 0.05).

Thus, the analysis based on the separation of the participants in two groups according to the cORP changes after exercise showed that in the low cORP group there was a significant increase in TBARS levels at 48 h and 72 h after exercise indicating an increase in lipid peroxidation (Figure 4). In our previous studies as well as in other studies increase in lipid peroxidation after eccentric exercise has also been reported [13, 15]. In the high cORP group, TBARS levels did not change significantly at any time-point after exercise suggesting an absence of lipid oxidation and maybe of oxidative stress. TAC did not change significantly in any of the two cORP groups. Actually, it would be expected for TAC to follow the decrease or increase of cORP, since both markers assess the total antioxidant capacity. However, the lack of consistency between them is probably, as mentioned above, due to the different methodologies used for their estimation.

In conclusion, in the present study, ORP markers were used for the first time for assessing eccentric exercise-induced oxidative stress. The results suggested that especially cORP marker may be used for predicting oxidative stress induced by eccentric exercise. That is, high decrease in cORP values after exercise compared to before exercise may indicate induction of oxidative stress. On the other hand, high increase in cORP values after exercise may indicate no existence of oxidative stress after eccentric exercise. These conclusions may help to identify eagerly individuals affected more by eccentric exercise-induced oxidative stress, since cORP measurement is easily and fast performed. This would allow appropriate interventions (e.g., antioxidant supplementation) to be applied for avoiding detrimental effects on health or shortening the recovery period in those individuals affected by oxidative stress. However, more studies, especially with larger samples, are needed in order to confirm these findings.

## Figures and Tables

**Table 1:** Delayed onset muscle soreness (DOMS) responses following the exercise session.

	Before	After	After 24 h	After 48 h	After 72 h
DOMSw	1.00 ± 0.00	2.63 ± 1.16	3.57 ± 1.16	4.63 ± 1.01	4.63 ± 1.34
DOMSsq	1.00 ± 0.00	3.21 ± 1.27	4.10 ± 1.10	5.00 ± 1.05	4.89 ± 1.10

^a^Values are the mean ± SD; DOMSw: DOMS assessed during walking; DOMSsq: DOMS assessed after performing a squat movement.

**Table 2:** Initial, 24 h, 48 h, and 72 h postexercise values of biomarkers when all participants were examined as one group (*n* = 19) (mean ± SD).

	Before	After 24 h	After 48 h	After 72 h	24 h % change	48 h % change	72 h % change
sORP (mV)	136.1 ± 13.2 (118.9–157.3)	135.5 ± 13.7 (156.4–116)	135.1 ± 16.2 (162.7–112.4)	134.8 ± 16.3 (159.4–104.2)	−0.45 ± 3.76 (−7.54–6.93)	−0.81 ± 5.57 (−12.60–7.45)	−0.97 ± 7.30 (−18.74–9.47)

cORP (*μ*C)	1.05 ± 0.71 (0.64–2.63)	1.08 ± 0.93 (0.34–4.07)	1.17 ± 0.89 (0.31–3.85)	1.19 ± 1.09 (0.32–4.75)	3.37 ± 35.27 (−47.53–87.56)	17.71 ± 52.62 (−38.40–150.00)	15.42 ± 52.16 (−56.27–118.89)

TAC (mmol DPPH/L plasma)	0.94 ± 0.09 (0.70–1.03)	0.99 ± 0.12 (0.70–1.22)	1.00 ± 0.16 (0.70–1.35)	1.04 ± 0.12 (0.89–1.35)	6.05 ± 14.20 (−19.67–45.09)	6.72 ± 15.82 (−11.79–41.83)	12.18 ± 17.30 (−7.15–45.61)

GSH (*μ*mol/g Hb)	3.09 ± 1.75 (0.42–5.60)	3.28 ± 1.70 (0.34–6.20)	3.04 ± 1.60 (0.55–6.00)	2.88 ± 1.51 (0.73–6.10)	15.26 ± 39.99 (−25.78–126.94)	10.54 ± 44.81 (−34.21–150.96)	8.03 ± 48.54 (−32.73–133.83)

Protein carbonyls (nmol/mg protein)	0.70 ± 0.18 (0.29–0.95)	0.74 ± 0.21 (0.38–1.07)	0.75 ± 0.19 (0.43–1.11)	0.76 ± 0.24 (0.43–1.48)	8.21 ± 23.82 (−36.96–57.78)	9.94 ± 26.39 (−35.92–63.16)	12.43 ± 36.8 (−25.17–128.00)

TBARS (*μ*mol/L)	6.7 ± 2.8 (3.4–13.0)	7.5 ± 2.8 (4.0–12.4)	7.4 ± 3.0 (3.1–13.0)	7.9 ± 2.9 (2.9–13.5)	16.95 ± 43.06 (−32.75–178.36)	17.09 ± 37.38 (−72.51–129.85)	27.08 ± 43.46 (−74.40–138.84)

Catalase (U/mg Hb)	151.9 ± 47.6 (92.8–310.4)	154.6 ± 27.0 (114.2–207.2)	142.3 ± 19.3 (105.9–169.9)	137.9 ± 18.6 (108.9–182.4)	9.98 ± 20.06 (−33.24–48.67)	−0.38 ± 26.10 (−45.25–56.04)	−2.18 ± 30.96 (−53.31–84.96)

The numbers in brackets show the minimum and maximum values.
